# Role of Direct Antiviral Agents in Treatment of Chronic Hepatitis C Infection in Renal Transplant Recipients

**DOI:** 10.1155/2018/7579689

**Published:** 2018-03-28

**Authors:** Sourabh Sharma, Debabrata Mukherjee, Ranjith K. Nair, Bhaskar Datt, Ananth Rao

**Affiliations:** Army Hospital Research & Referral, Delhi, India

## Abstract

**Background:**

Since the introduction of direct antiviral agents (DAAs), morbidity of HCV has considerably decreased but still no guidelines have been formulated in renal transplant recipients (RTRs). We studied efficacy and tolerability of direct antiviral agents in RTRs.

**Methods:**

This prospective observational study was conducted at Army Hospital Research & Referral, Delhi, from June 2016 to May 2017. Forty-five HCV infected RTRs with stable graft function were included.

**Results:**

Median time between renal transplantation and the start of anti-HCV therapy was 36 months (1–120 months). The majority (66.7%) were infected with genotype 3. Baseline median HCV RNA level was 542648 IU/ml (1189–55028534 IU/ml). Sofosbuvir-Ribavirin combination (24 weeks) was given to 30 patients including 3 cirrhotics, Ledipasvir-Sofosbuvir combination to 8 patients, and Daclatasvir-Sofosbuvir combination to 7 patients, including 2 cirrhotics. Rapid virological response was observed in 29 patients treated with Sofosbuvir/Ribavirin, all 8 patients on Sofosbuvir/Ledipasvir, and all 7 patients on Sofosbuvir/Daclatasvir. End treatment response and sustained virological response (12 weeks) were achieved in all patients irrespective of genotype or treatment regimen. Decrease in mean HCV RNA level and transaminase level was statistically significant (*p* < 0.01). Ribavirin was significantly associated with anaemia (*p* = 0.032).

**Conclusions:**

DAA regimens are well tolerated and highly efficacious. Response to DAA is good irrespective of genotype, drug combination, initial HCV RNA level, age or sex of patient, or graft age. However, Sofosbuvir/Ledipasvir and Sofosbuvir/Daclatasvir combination is preferable.

## 1. Introduction

Chronic hepatitis C virus (HCV) infection remains an important health problem, which is associated with deleterious consequences in renal transplant recipients [1–4]. Besides hepatic complications, several extrahepatic complications contribute to reduced patient and allograft survival in HCV infected renal transplant recipients (RTRs) [5–9]. HCV infection is associated with an increased risk of mortality in these patients as a consequence of liver disease, higher infection rates, and cardiovascular disease [1, 6, 9]. Moreover, HCV infection in RTR is an independent risk factor for graft loss, and it is associated with proteinuria, chronic rejection, transplant glomerulopathy, posttransplant diabetes, and HCV associated glomerulonephritis [7, 9–11].

However, HCV infection should not be considered as a contraindication for renal transplantation because patient survival is better with transplantation than on dialysis. Until recently, treatment of HCV infection was IFN*α* based [12], which had been associated with higher renal allograft rejection rates [13, 14] and very modest success in viral eradication [12, 15]. Therefore, IFN*α* therapy is preferable before transplantation and is presently recommended only in rapid worsening hepatic injury, like in Fibrosing cholestatic hepatitis [16, 17] or in life-threatening vasculitis [16] when potential benefits outweigh risks in cases of life-threatening liver injury [18]. IFN-free treatment regimens, like direct antiviral agents (DAAs), because of their greater efficacy, reduced toxicity, and minimal interaction with immune-suppressants currently represent promising and attractive therapeutic options. The efficacy of these oral agents used with Ribavirin or in combination with one another yields a sustained virological response at 12 weeks of greater than 90% among patients who are treatment naïve [19]. However, the majority of initial clinical trials for the DAAs have excluded RTR or patients with chronic kidney disease with eGFR less than 30 ml/min, including those on hemodialysis. Though the efficacy of DAAs in liver-transplant recipients had been established in studies done by Forns et al. [20] and Charlton et al. [21] in 2015, published data on safety and efficacy of DAAs in RTR is scarce. Clinical trials are required to closely evaluate these regimens in RTRs. There is also a need for further studies to determine optimal immunosuppressive regimens after transplantation in HCV infected recipients.

## 2. Materials and Methods

### 2.1. Setting

This study was conducted from June 2015 to May 2017 at Department of Nephrology, Army Hospital Research & Referral, New Delhi.

### 2.2. Study Design

This is a single-center prospective observational study. Renal transplant recipients at least one month after transplant with replicating HCV infection and fulfilling the inclusion criteria were enrolled after informed written consent. The study was approved by institutional ethical committee and institutional scientific committee.

### 2.3. Sample Size

In this study, DAA therapy was assumed to be effective with SVR 24 > 90% in HCV infected RTR [22] compared to interferon based regimen in which SVR 24 used to be achieved in 50% of the cases [12]

(1) p1=0.5;p2=0.9.

Effect size, that is, difference between proportions (*p*1 − *p*2), = 0.5 − 0.9 = −0.4.

(2) $$\begin{array}{c} \text{Pooled prevalence}=\frac{p1+p2}{2}=0.7. \end{array}$$

So,

(3) $$\begin{array}{c} \text{sample size}=\frac{2{\left(1.96+0.84\right)}^{2}0.4\left(1-0.4\right)}{{\left(-0.4\right)}^{2}}=24. \end{array}$$

It was planned to include at least thirty patients in the study. However, finally forty-five patients were recruited for the study.

### 2.4. Inclusion Criteria

- Renal transplant recipient ≥ 18 years of age, at least 1 month after transplant surgery
- Stable graft function with eGFR ≥ 30 mL/min/1.73 m^2^ as estimated by MDRD study equation
- Replicating HCV infection (detectable HCV RNA by quantitative PCR)
- Absolute neutrophil count ≥ 750 cells/mm^3^; platelet count ≥ 50,000 cells/mm^3^; hemoglobin ≥ 11 g/dL for women and ≥12 g/dL for men

### 2.5. Exclusion Criteria

- Patients < 18 years of age
- Pregnant or nursing women
- Coexisting malignancy
- Coinfection with Human Immunodeficiency Virus or Hepatitis B Virus
- eGFR < 30 mL/min/1.73 m^2^ as estimated by MDRD study equation

### 2.6. Conduct of Study

At the initial visit, after informed written consent, all patients were subjected to a baseline hemogram, renal function tests (blood urea nitrogen and serum creatinine level), and liver function tests (serum AST and ALT levels). eGFR was calculated by MDRD study equation. Baseline CNI (Tacrolimus/Cyclosporine) levels were assessed by LC-MS/MS method. Pretreatment quantitative HCV RNA level was determined by HCV RT-PCR kit v1.0 (RealStar®, Altona Diagnostics GmbH). The concentration of Quantification Standards was given in IU per ml, corresponding to the concentration of purified nucleic acid. HCV genotype was determined using VERSANT HCV LiPA 2.0. Liver fibrosis was evaluated before therapy by measuring liver stiffness, using a Fibroscan according to the manufacturer's instructions (Fibroscan 402, Echosens, Paris, France). The results of elastometry were expressed in kilopascals, and the median value was considered representative of the elastic modulus of the liver. A liver stiffness of less than 7.4 kPa was considered to be equivalent to a METAVIR score of F0 to F2, a liver stiffness of 7.5–12.4 kPa was considered to be equivalent to a METAVIR score of F3, and cirrhosis (METAVIR F4) was defined by values of 12.5 kPa or greater [23].

All patients were treated with DAA therapy. Initially, only Sofosbuvir/Ribavirin combination was available in Indian market, so initially (2015) HCV genotype 1 infected patients were given Sofosbuvir 400 mg daily/weight based Ribavirin, adjusted for anaemia (*n* = 7). Subsequently, once Ledipasvir 90 mg/Sofosbuvir 400 mg and Daclatasvir 60 mg/Sofosbuvir 400 mg daily regimen became available (2016 onwards), newer regimen was given according to genotype. The types of DAA combinations and the duration of therapies according to each genotype are presented in Table 1.

Initial Ribavirin dose was weight based; patients < 60 Kg weight received 800 mg daily, patients between 60 and 74 Kg weight received 1000 mg daily, and patients with weight ≥ 75 Kg received 1200 mg daily dose. The dose was modified as per hemoglobin levels as follows: Hb < 10 gm/dl: 400 mg daily if weight < 60 Kg or 600 mg daily if weight ≥ 60 Kg, Hb < 8.5 gm/dl: Ribavirin discontinued and reintroduced once Hb > 8.5 gm%.

In all patients, hemogram, kidney function tests, transaminase levels, and virological parameters were assessed before therapy, at 4 weeks, 12 weeks, and 24 weeks during the therapy, and at 12 weeks after completion of therapy. Adherence to the treatment was determined by pill counts at each visit and patient interviews.

### 2.7. Outcomes

The primary efficacy end point was fall in HCV RNA level to less than 25 IU/mL at week 12 or 24 of therapy (end of treatment response, ETR) while the secondary efficacy end point was achievement of SVR12 (sustained virological response after 12 weeks of completion of therapy) [28, 29].

Rapid virological response (RVR) is defined as undetectable HCV RNA using a sensitive PCR assay at week 4 of therapy, and early virological response (EVR) is defined as undetectable HCV RNA at week 12 of therapy [28, 29].

The safety end points included the rate of adverse events and rate of discontinuation of DAA therapy in the HCV infected RTRs.

### 2.8. Statistical Evaluation

The data so collected were entered in MS Excel. Results are expressed as mean ± standard deviation and median. Comparison of continuous and categorical variables was done by student *t*-test and chi-square test, respectively. The level of significance was defined as *p* < 0.05. SPSS® (Statistical Package for the Social Sciences) version 22 Statistics for Windows (IBM® Corp, Armonk, NY) was used for data analysis.

## 3. Results

### 3.1. Baseline Patient Characteristics

Forty-five RTRs were included in the study and received DAA therapy. Baseline patient demographic and clinical characteristics are depicted in Table 2.

### 3.2. HCV Genotype and Viral Load at Baseline

HCV genotype and viral load at baseline are shown in Table 2.

### 3.3. DAA Regimens

**Figure 1:**
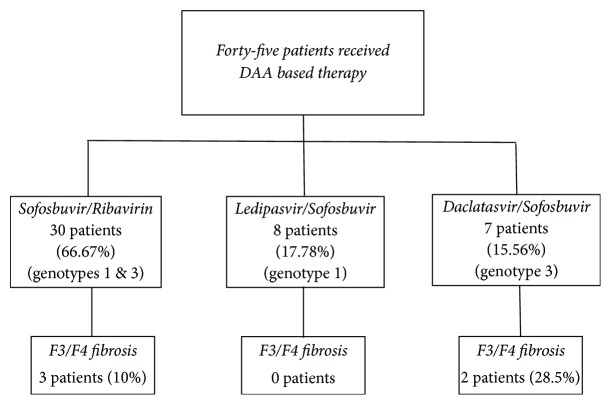
Study population: DAA regimen stratification of study population.

All patients were treated with DAA therapy. DAA regimen given to the study population is as depicted in Figure 1. Advanced fibrosis stage (F3) was seen in 2 (4.4%) patients and cirrhosis (F4) was present at baseline in 5 (11.1%). Thirty-eight (84.4%) RTRs had equivalent METAVIR score of F0 to F2. Out of the five patients with cirrhosis, none had hepatic decompensation.

### 3.4. Patients with Cirrhosis

There were five patients with compensated cirrhosis and all of them were old transplant patients. One patient, infected with genotype 1, was treated with Sofosbuvir/Ribavirin for 24 weeks. Out of the remaining four cirrhotic cases with genotype 3 infection, two were treated with Sofosbuvir/Ribavirin for 24 weeks and two with Daclatasvir/Sofosbuvir regimen for 24 weeks.

### 3.5. Virological Response

Response of the patients to DAA therapy is depicted in Table 3.

- Rapid virological response (RVR): RVR was observed in 29 of the 30 patients treated with Sofosbuvir/Ribavirin. All 8 patients on Sofosbuvir/Ledipasvir and all 7 patients on Sofosbuvir/Daclatasvir showed RVR.
- Early virological response (EVR): EVR was observed in 29 of the 30 patients (96.67%) treated with Sofosbuvir/Ribavirin. One patient (HCV genotype 3) failed to achieve EVR but there was substantial decline in HCV RNA level from 1020062 IU/ml to 460 IU/ml after 12 weeks of therapy.
- End treatment response (ETR): one of the patients included in this study, who was on Sofosbuvir/Ribavirin, expired during study due to Nocardiosis, after achieving EVR. End of treatment response (ETR) was achieved in all 29 remaining patients treated with Sofosbuvir/Ribavirin including one who had not achieved EVR. All 8 patients with Sofosbuvir/Ledipasvir and 7 patients with Sofosbuvir/Daclatasvir achieved ETR. Hence, all patients achieved ETR irrespective of the genotype or treatment regimen used.
- Sustained virological response at 12 weeks (SVR 12): overall, all patients included in the study (excluding one expired) achieved SVR 12 irrespective of genotype or treatment regimen. No patient experienced relapse during therapy.

**Figure 2:**
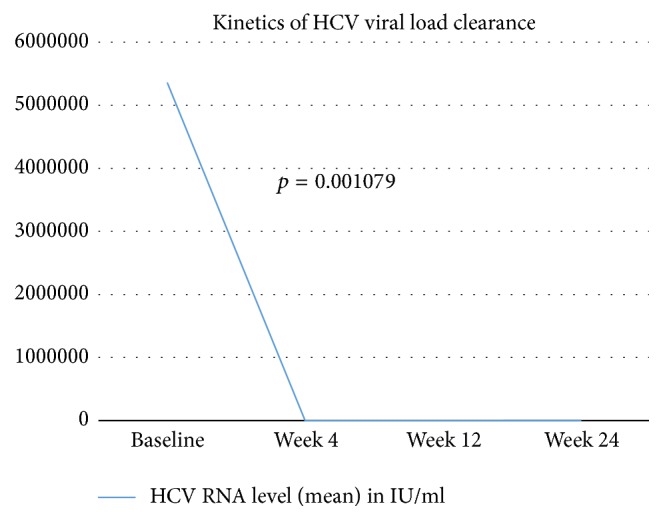
Kinetics of HCV viral load clearance. The decrease in mean HCV RNA level from baseline at start of therapy to that at week 4 was statistically significant (two-sample *t*-test; *p* = 0.001079).

The kinetics of HCV viral load clearance have been depicted in Figure 2.

### 3.6. Liver Function

**Figure 3:**
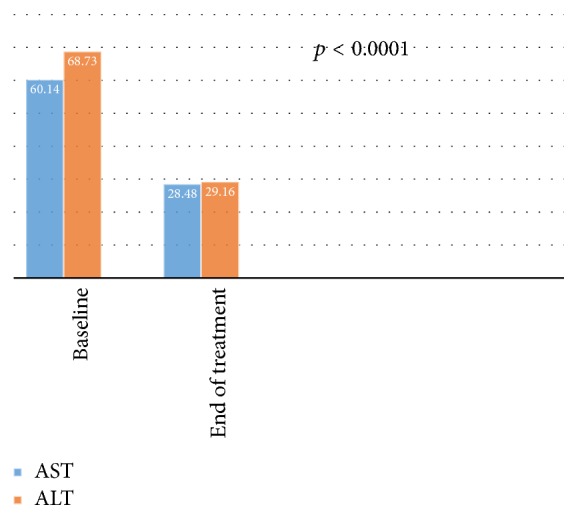
Outcome of serum transaminase level before and after DAA therapy (two-sample *t*-test; *p* < 0.0001).

Serum AST/ALT levels decreased significantly (*p* < 0.0001) following DAA therapy (Figure 3).

### 3.7. Renal Allograft Function

At the initiation of antiviral therapy, all patients had a GFR of 30 ml/min or greater. Twenty-one patients (46.67%) had a GFR of 60 ml/min or greater, 12 patients (26.67%) had a GFR between 45 and 59 ml/min, and 12 patients (26.67%) had a GFR between 30 and 44 ml/min. During therapy, overall no significant change in graft function was observed.

Two (4.44%) patients experienced an increase in serum creatinine > 25% during treatment. Out of these two, one patient had developed diarrhoea and as it improved, the graft function recovered spontaneously. The other patient underwent graft biopsy showing an antibody mediated rejection, which was treated with plasmapheresis, IVIg, and Bortezomib. His graft function improved but did not touch the baseline. Tacrolimus level of first patient was 5.8 ng/ml (4 years after transplant) while that of second patient was 9.8 ng/ml (1 month after transplant), which were well within therapeutic range.

### 3.8. Adverse Events

The Sofosbuvir/Daclatasvir and Sofosbuvir/Ledipasvir regimen were well tolerated with least reported adverse events. Adverse events are shown in Table 4.

### 3.9. Hematological Tolerance

As depicted in Table 5, Ribavirin was significantly associated with anaemia (*p* = 0.032). Ribavirin dose reduction was required in nine patients (81.82%), out of which two patients (18.18%) required discontinuation of Ribavirin. Recombinant erythropoietin support or blood transfusion was not required in any patient. No patient discontinued therapy due to adverse events related to DAA therapy.

### 3.10. Immunosuppression

All of the forty-five patients were on triple drug immunosuppression. Thirty-nine patients (86.67%) were on Tacrolimus based immunosuppressive regimen and four (8.89%) were on Cyclosporine based regimen while two (4.44%) were on Everolimus based regimen. The most common combination therapy was Tacrolimus, Mycophenolate Mofetil, and Prednisolone. All patients had stable CNI (Tacrolimus/Cyclosporine) trough levels during DAA therapy. No change in immunosuppression was made during DAA therapy or within 12 weeks of completion of therapy.

## 4. Discussion

HCV infection remains an important health problem in the hemodialysis and renal transplant population and is associated with deleterious consequences. Until recently, treatment of HCV infection was IFN*α* based, which has been associated with poor efficacy, poor compliance due to adverse effects, and higher renal allograft rejection rates. DAAs represent promising and attractive therapeutic options because of their greater efficacy, reduced toxicity, and minimal interaction with immunosuppressants. However, data in RTRs is scarce and yet there is no approved therapy or guideline for use of DAA in this population. At the time study was initiated (June 2015), there were no published data on use of DAAs in RTRs. Four studies were published on this subject over the last two years [24–27]. Our aim was to study the efficacy of DAA in HCV positive RTRs in achieving ETR and SVR. In this single-center prospective observational study, conducted between Jun 2015 and May 2017, we describe our center's experience in treating HCV infected RTR with DAA regimen.

Forty-five patients received DAA therapy in our study and this sample size is highest among prospective single-center studies published so far. The study pattern and demographics of different published studies have been compared with our study in Table 6.

The study population in our center was younger as compared to other studies which can be explained by fact that the majority of the patients treated for ESRD at this center are younger, serving Armed Forces personnel or their family member. Forty-nine percent of patients in our study were women unlike other studies where the majority of patients were men.

The median time between renal transplantation and start of anti-HCV therapy was shorter as compared to other studies. Nine HCV infected patients were started on DAA therapy after one month of transplantation once graft function stabilized. This is the reason for shorter median transplant-to-treatment time in our study.

Forty percent of patients (18/45) had documented HCV infection prior to transplantation, while 27 patients were detected to have HCV infection after undergoing renal transplantation. Lin et al. [26] have described 88% of the patients being documented to have HCV infection prior to transplantation. In our study, the majority of patients were diagnosed with HCV infection after transplantation, possibly because, at our center, HCV RNA PCR is not done routinely prior to transplantation; pretransplant HCV screening is based upon presence of anti-HCV antibodies in serum which can be negative in early infection. Also, detection of anti-HCV antibodies by third-generation enzyme-immunoassay allows false negative results in dialysis patients [30, 31].

In this study, median serum creatinine at HCV treatment initiation was 1.22 mg/dl and the median eGFR was 57 mL/min/1.73 m^2^. These values are comparable to that described by Lin et al. [26] in their study (median baseline serum creatinine 1.21 mg/dl and mean baseline eGFR 70.9 ml/min/1.73 m^2^) but lower than that reported by Fernández et al. [27] who had included patients with eGFR < 30 ml/min/1.73 m^2^ in their study.

Genotype 3 was the most prevalent genotype in our patient population followed by genotype 1. As depicted in Table 6, in other studies done in USA, France, and Spain, genotype 1 was the predominant HCV infection in RTRs. This is in concordance with studies which show that HCV genotype 3 is most prevalent in India while genotype 1 is more prevalent in Europe and Americas [32, 33].

In our study, rapid virological response (RVR) was observed in 97.78%. One patient had expired during study due to Nocardiosis, after achieving EVR. End treatment response (ETR) and SVR 12 were 100% among the remaining 44 patients and none of them experienced relapse. We did not observe any difference in rate of SVR12 in patients receiving 12 weeks of therapy without Ribavirin or 24 weeks of therapy with Ribavirin. We observed that DAA is highly effective in patients with cirrhosis, though all patients with cirrhosis were treated with DAA therapy for 24 weeks.

As depicted in Table 6, in the studies of Kamar et al. [24] and Sawinski et al. [25], SVR 12 was noted in 100% of the cases while Ladino et al. [11] and Fernández et al. [27] achieved SVR 12 in 91% and 98% of the cases, respectively. We observed that there was no impact on response by HCV genotype, initial HCV RNA level, age or sex of the patient, or age of the graft. Also, there is no difference in response to therapy on the basis of timing of DAA initiation; those treated within the first 6 months after transplantation cleared the virus as easily as those treated later after transplantation.

Serum AST/ALT levels normalized after DAA therapy (*p* < 0.0001) in our study. Kamar et al. [24] had also shown a significant decline in transaminase levels in their study. Similarly, Sawinski et al. [25] and Lin et al. [26] have also shown a declining trend in their respective studies.

Overall, DAA therapy was well tolerated with no significant impact on graft function. Two patients experienced an increase in serum creatinine > 25% during treatment. Out of these two, one patient recovered spontaneously while the other patient underwent graft biopsy which showed an antibody mediated rejection. Lin et al. [26] and Fernández et al. [27] have also reported rejection while on DAA therapy for which Kamar et al. [24] and Lin et al. [26] have postulated that this might be related to increased hepatic metabolism of CNI as a consequence of improvement in liver function following viral eradication. Kamar et al. [24] and Lin et al. [26] had reported no significant change in graft function during DAA therapy. No rejection or graft loss was observed in their study. Sawinski et al. [25] also had similar results but have reported rise in serum creatinine by >0.25 mg/dL in four patients. This rise was attributed to supratherapeutic Tacrolimus levels in two patients. Fernández et al. [27] did not find any significant change in serum creatinine or eGFR in their study but reported increase in serum creatinine > 25% in seventeen (16%) patients.

We observed that there was no incidence of discontinuation of DAA therapy because of significant adverse effect. Ribavirin was significantly associated with anaemia (chi-square test; *p* = 0.032). This observation is supported by the study done by Fernández et al. [27] in which they have reported association between Ribavirin use and anaemia. Fourteen out of 42 cases on Ribavirin (33%) developed anaemia requiring dose adjustment in 13 (31%) and discontinuation in 8 (19%) cases. Six cases (14%) received a blood transfusion. Sawinski et al. [25] have also reported good tolerability to DAA therapy in their study. In their study, two out of three patients treated with Ribavirin (66.67%) required dose reduction due to anaemia, one of whom required a blood transfusion. Lin et al. [26] reported 11 patients (46%) to have adverse events during DAA therapy. One patient developed serious adverse event while on Sofosbuvir/Simeprevir in form of sinus bradycardia and junctional escape rhythm. Patients on Ribavirin experienced more adverse events as compared to those not on Ribavirin. Two out of seven patients on Ribavirin had to discontinue Ribavirin because of shortness of breath, fatigue, and gout flare while two patients (28.57%) developed anaemia which recovered after treatment.

We observed that all patients in our study had stable CNI trough levels during DAA therapy. No change in immune-suppression was made during DAA therapy or within 12 weeks of completion of therapy. This finding is supported by study done by Lin et al. [26] in which majority of patients had stable CNI trough levels during DAA therapy. Kamar et al. [24] also reported no significant change in dose during DAA therapy. However, after HCV clearance, there was decrease in Tacrolimus trough levels, whereas Tacrolimus dose remained unchanged. Sawinski et al. [25] reported that almost half (45%) of the patients required dose adjustment of their CNI during therapy though this was not significantly associated with a particular regimen (*p* = 0.84). Similarly, in a study done by Fernández et al. [27], Tacrolimus dose adjustment was required in 47 out of 75 patients (62.6%). This was not significantly associated with a particular regimen of DAAs (*p* > 0.05). Recently, Fernández-Ruiz et al. [34] reported that 80.6% of the cases on Tacrolimus required dose adjustment while on DAA therapy to maintain desired levels. In their study, graft function remained stable while on therapy but significant decrease in graft function was observed (*p* value < 0.001) throughout the first 12 months after the end of therapy. This study highlights the role of continuous monitoring of drug levels even after completion of DAA regimen.

There were certain limitations in our study. Our follow-up period in the patients inducted later into the study was necessarily shorter than that for patients included in the earlier part of study, though all patients were followed up for 12 weeks after completion of therapy. The number of patients treated with Ledipasvir/Sofosbuvir and Daclatasvir/Sofosbuvir was lower than those put on Sofosbuvir/Ribavirin due to recent availability of these drugs in India. Since HCV RNA PCR is not done routinely during pretransplant evaluation at our center, 60% of patients were detected to have HCV infection only in posttransplant period.

## 5. Conclusions

We conclude by our study that all-oral, interferon-free DAA regimens are well tolerated and are highly efficacious, with an SVR12 rate of 100% among a heterogeneous and complex renal transplant population with HCV infection. Response to DAA was good irrespective of genotype, drug combination, initial HCV RNA level, age or sex of the patient, or age of the graft. However, Sofosbuvir/Ledipasvir and Sofosbuvir/Daclatasvir combination is the preferred drug combination in genotype 1 and genotype 3, respectively, as they were tolerated better compared to DAA with Ribavirin.

There is no difference in response to therapy on the basis of timing of DAA initiation. Patients who were treated within the first 6 months after transplantation cleared the virus as easily as those treated later after transplantation. DAA is highly effective in patients with cirrhosis when treated for 24 weeks. Hence, HCV positive ESRD patients with compensated cirrhosis should not be denied transplant.

The high efficacy and tolerability of DAA hold great promise for renal transplant population in improving their outcome. Chronic HCV infected patients awaiting renal transplantation can be safely transplanted and then initiated on DAAs once their eGFR rises above 30 mL/min/1.73 m^2^.

## Figures and Tables

**Table 1:** Combination of DAAs and treatment according to genotype and DAA availability.

HCV genotype	*N*	DAA combination	Duration (weeks)
1	7	Sofosbuvir + Ribavirin	24
	8	Ledipasvir + Sofosbuvir	12

3	23	Sofosbuvir + Ribavirin	24
	7	Daclatasvir + Sofosbuvir	12 (24 in cirrhotic; *n* = 2)

**Table 2:** Demographic and clinical characteristics at baseline.

Characteristics	Overall subjects (*n* = 45)
Recipient age (years), median (range)	38 (23–68)
Gender, *n* (%)	
Male	23 (51.1%)
Female	22 (48.9%)
Time to initiation of therapy after transplantation (months), median (range)	36 (1–120)
Patients detected HCV positive, *n* (%)	
Before transplantation	18 (40%)
After transplantation	27 (60%)
Diabetes mellitus, *n* (%)	4 (8.9%)
New onset diabetes after transplantation, *n* (%)	8 (17.8%)
HCV genotype, *n* (%)	
1	15/45 (33.3%)
3	30/45 (66.7%)
2, 4, 6	Nil
METAVIR fibrosis stage, *n* (%)	
F0–F2	38 (84.4%)
F3-F4	7 (15.6%)
Hepatic decompensation	Nil
HCV viral load (IU/ml), median (range)	542648 (1189–55028534)
Serum creatinine at treatment initiation (mg/dl), median (range)	1.22 (0.66–2.0)
Estimated GFR (ml/min/1.73 m^2^), median (range)	57 (30–118)
Baseline immunosuppression regimen (in combination with Mycophenolate Mofetil/Azathioprine and Prednisolone), *n* (%)	
Tacrolimus based	39 (86.7%)
Cyclosporine based	4 (8.9%)
Everolimus based	2 (4.4%)

**Table 3:** Virological response to DAA therapy.

Virological response	Sofosbuvir/Ribavirin (*n* = 30)	Sofosbuvir/Daclatasvir (*n* = 7)	Sofosbuvir/Ledipasvir (*n* = 8)	Total (*n* = 45)
RVR	29	7	8	44
ETR	29	7	8	44
SVR12	29	7	8	44

**Table 4:** Adverse events reported while on DAA therapy.

Adverse events	Sofosbuvir/Ribavirin (*n* = 30)	Sofosbuvir/Daclatasvir (*n* = 7)	Sofosbuvir/Ledipasvir (*n* = 8)	Total (*n* = 45)
Anaemia	11	1	0	12
Diarrhoea	9	2	1	12
Headache	6	1	3	10
Nausea	4	4	2	10
Leucopenia	3	0	0	3
Thrombocytopenia	3	0	0	3
Influenza-like illness	2	0	0	2
Myalgia	1	1	0	2
Graft dysfunction	1	0	1	2

**Table 5:** Hematological tolerance. Association of anemia with Ribavirin.

	Patients on DAA with Ribavirin	Patients on DAA without Ribavirin	*p* value (chi-square test)
Anaemia	11	1	0.03193 (chi-square statistic 4.6023)
Ribavirin dose reduction	9	0	
Ribavirin discontinuation	2	0	
EPO/transfusion	0	0	

**Table 6:** Comparison of study pattern, demographics, virological profile, and response to therapy with other published studies.

	Kamar et al. [24]	Sawinski et al. [25]	Lin et al. [26]	Fernández et al. [27]	Present study
Study period	2014-2015	2014-2015	2013–2015	2015-2016	2015–2017
Study design	Prospective	Prospective	Retrospective	Retrospective	Prospective
Study center	Single-center	Single-center	Multicenter	Multicenter, national	Single-center
Place of study	*France*	*USA*	*USA*	*Spain*	*India*
Sample size	25	20	24	103	45
Age in years	54 ± 10	57 ± 5.5	60 (34–70)	55 (27–74)	38 (23–68)
M : F	15 : 10	16 : 4	19 : 5	69 : 34	23 : 22
Time since renal transplantation	146 months (1–329)	888 days (341–1621)	96 months (2–492)	147 months (1–561)	36 months (1–120)
Genotype:					
1	19	17	21	85	15
3	1	0	0	7	30
2, 4, 6	5	3	3	10	0
HCV RNA level	Mean 6.33 ± 0.6 log IU/ml	Median 6.5 log IU/ml (range 6.3–7 log IU/ml)	Median 1,922,552 IU/ml (1060–22,600,000 IU/ml)	Median 6.61 log IU/ml (2.87–7.79 log IU/ml)	Median 5,42,648 IU/ml (range 1189–55,028,534 IU/ml)
Virological response:					
RVR	88% (22/25)	-	-	59%	97.78%
ETR	100%	95%	83.33%	100%	97.78%
SVR 12	100%	100%	91%	98%	100%
Immunosuppression:					
Tacrolimus based	19 (76%)	19 (95%)	19 (79.16%)	75 (72.82%)	39 (86.67%)
Cyclosporine based	5 (20%)	1 (5%)	3 (12.5%)	12 (11.65%)	4 (8.89%)
mTOR inhibitor based	1 (4%)	0	1 (4.16%)	7 (6.8%)	2 (4.44%)

## Abbreviations

ALT: Alanine aminotransferase
AST: Aspartate aminotransferase
BUN: Blood urea nitrogen
CNI: Calcineurin inhibitor
DAA: Direct antiviral agents
eGFR: Estimated glomerular filtration rate
ESRD: End stage renal disease
ETR: End of treatment response
EVR: End virological response
GFR: Glomerular filtration rate
Hb: Hemoglobin
HCV: Hepatitis C virus
IFN: Interferon
IFN*α*: Interferon alpha
IU: International unit
LC-MS/MS: Liquid chromatography-mass spectrometry
MDRD: Modification of diet in renal disease
MMF: Mycophenolate Mofetil
PCR: Polymerase chain reaction
RNA: Ribonucleic acid
RTR: Renal transplant recipients
RVR: Rapid virological response
SVR12: Sustained virological response at 12 weeks (of completion of treatment)
SVR24: Sustained virological response at 24 weeks
TLC: Total leucocyte count.
